# Effect of the Citrus Lycopene β-Cyclase Transgene on Carotenoid Metabolism in Transgenic Tomato Fruits

**DOI:** 10.1371/journal.pone.0032221

**Published:** 2012-02-24

**Authors:** Fei Guo, Wenjing Zhou, Jiancheng Zhang, Qiang Xu, Xiuxin Deng

**Affiliations:** 1 Key Laboratory of Horticultural Plant Biology of Ministry of Education, Huazhong Agricultural University, Wuhan, China; 2 National Key Laboratory of Crop Genetic Improvement, Huazhong Agricultural University, Wuhan, China; United States Department of Agriculture, United States of America

## Abstract

Lycopene β-cyclase (LYCB) is the key enzyme for the synthesis of β-carotene, a valuable component of the human diet. In this study, tomato constitutively express *Lycb-1* was engineered. The β-carotene level of transformant increased 4.1 fold, and the total carotenoid content increased by 30% in the fruits. In the transgenic line, the downstream α-branch metabolic fluxes were repressed during the three developmental stages while α-carotene content increased in the ripe stage. Microarray analysis in the ripe stage revealed that the constitutive expression of *Lycb-1* affected a number of pathways including the synthesis of fatty acids, flavonoids and phenylpropanoids, the degradation of limonene and pinene, starch and sucrose metabolism and photosynthesis. This study provided insight into the regulatory effect of *Lycb-1* gene on plant carotenoid metabolism and fruit transcriptome.

## Introduction

Carotenoids represent a highly diverse classes of natural pigments, with >700 naturally occurring molecules of this type already catalogued [1]. Plant carotenoids are composed of a C40 isoprenoid skeleton to which are attached polyene chains containing up to 15 conjugated double bonds. Plant carotenoids are synthesized by nuclear-encoded enzymes in the plastids via the MEP pathway [2]. Their in planta function is to protect photosynthesis from damage caused by excessive light [3], [4] and to provide a substrate for the synthesis of certain phytohormones [5], [6]. They also serve as precursors for the synthesis of a number of biologically important compounds [7], [8], [9], [10]. Some carotenoids are exploited as natural food colorants, and others are used in the cosmetics and pharmaceutical industries [11]. They also represent an important component in human nutrition, providing both a source of provitamin A and some protection against various cancers [12].

Carotenoid synthesis is a complex secondary metabolic system [13]. In the tomato fruit, LYCB activity yields β-carotene [14], which is a major source of vitamin A for the human diet [12], [15]. The human body is unable to synthesize carotenoids, instead has to rely on their presence in the diet. As tomato fruits are rich in β-carotene precursors, there is a substantial interest in engineering carotenoid synthesis in tomato with a view to enhancing its β-carotene content. The carotenoid content of the tomato fruit has been successfully manipulated via a number of transgenic strategies. Plants expressing a *Erwinia uredovora* phytoene desaturase (*crtI*) gene, the product of which converts phytoene into lycopene, was successful in increasing fruit β-carotene content by three fold [16]. Similarly, the heterologous expression in the tomato fruit of *crtB*, an *Erwinia uredovora* phytoene synthase gene, raised it by between two and four fold, divided between phytoene, lycopene, β-carotene and lutein [17]. Plants over-expressing endogenous *Lycb* also produce fruit having an enhanced content of β-carotene as a result of the almost complete cyclization of lycopene [18]; meanwhile the down-regulation of DE-ETIOLATED1 (*Tdet1*) had a similar result [19]. The plastidial expression of a bacterial *Lycb* gene was able to trigger the conversion of lycopene to β-carotene, resulting in a four fold enhancement in the fruit's pro-vitamin A content [20]. Genes encoding LYCB either from *Erwinia herbicola* or the daffodil (*Narcissus pseudonarcissus*) have also been introduced into tomato plastids. While expression of the former appeared to have little effect on carotenoid composition, the product of the latter was able to efficiently convert lycopene into β-carotene [21].

The regulation of β-carotene synthesis has been widely researched. In wild type tomatoes, the B gene, which encodes a form of LYCB, is only expressed at low levels during the breaker stage of ripening, but in the *Beta* mutant, its transcription is substantially enhanced, resulting in a notably higher accumulation of β-carotene [14]. In cauliflower, a mutation in *Or* results in the deposition of substantial quantities of β-carotene in various tissues [22]. Meanwhile in maize, it has been demonstrated that the β-carotene hydroxylase 1 gene (*crtRB1*) is responsible for a major proportion of the variation in β-carotene concentration and conversion in the grain [23]. Nevertheless, the consequences of overproducing β-carotene, both with respect to carotenoid synthesis and to the transcriptome as a whole have not been widely explored. In this study, we have explored the effect on carotenoid synthesis of constitutively expressing a citrus *Lycb-1* gene in tomato. We have compared gene expression profiles in the fruit of wild type and the transgenic plants at a variety of developmental stages, which has allowed us to evaluate the effect of the over-production of β-carotene on the expression of other genes involved in carotenoid synthesis, and on the transcriptome in the ripe stage as a whole.

## Results

### Transgenic tomato constitutively expressing Lycb-1

Of the 14 presumptive primary transformants regenerated by agrobacterium transformation method, 9 were proved positive by both PCR and Southern hybridization. The fruit color by these transformants ranged from yellow to red, with four plants producing clearly yellow fruit. The predominant carotenoid responsible for this changed pigmentation was β-carotene as revealed by HPLC analysis. Southern blotting revealed that the copy number of *npt*II in these lines ranged from one to three (Fig. S1). Single copy carrier progeny of Lycb1-T_0_17 and -T_0_30 were self-fertilized to generate fixed lines, as validated using a PCR-based progeny test. The effect of constitutive *Lycb-1* expression during fruit development and ripening was tested in Lycb1-T_0_17 progeny over three generations, and the same line was also used to study the effect of constitutive *Lycb-1* expression on the transcriptome as a whole.

### Changes in the carotenoid, sugar and ABA content of plants constitutively expressing Lycb-1

**Figure 1:**
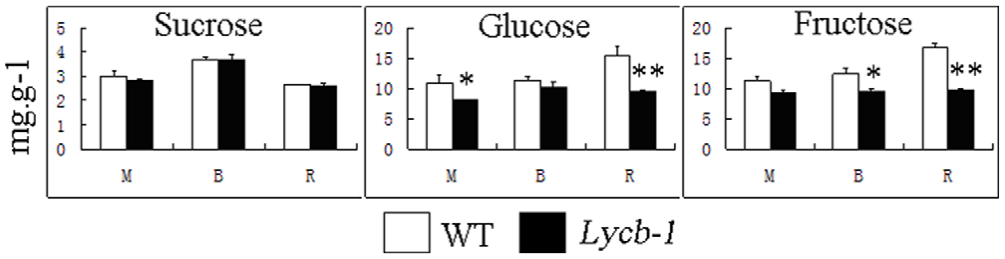
Soluble sugar (fructose, glucose and sucrose) contents in the fruits of Wild-Type and *Lycb-1* plants. Columns and bars represent, respectively, the mean and SD (n = 3), Student's t tests illustrate statistical significance (* P<0.05, ** P<0.01).M: mature green, B: breaker, R: ripe.

The levels of the predominant carotenoids at the mature green, breaker and ripe stages of *Lycb-1* and WT fruit are shown in Table 1. At the mature green stage, the only detected carotenoids were lutein and β-carotene. The content of the former in WT was 1.1 fold higher than in *Lycb-1*, while for the latter, the transgenic line contained 0.2 fold more than the WT. At the breaker stage, lycopene was present in WT, but not in *Lycb-1* fruit. The phytoene and lutein content of the WT fruit was, respectively, 1.6 and 1.0 fold higher than that of the *Lycb-1* fruit, while the β-carotene content was 0.5 fold higher in the transgenic fruit. At the ripe stage, β-carotene persisted as the principal carotenoid in the transgenic fruit, whereas in the WT fruit, its place was taken by lycopene. The *Lycb-1* fruit accumulated β-carotene up to 1.1 mg/gDW (dry weight). This increase of β-carotene greatly exceeded the extent of the decrease in lycopene, resulting in a fruit with a 30% higher content of carotenoid than the WT. *Lycb-1* fruits also accumulated substantial quantities of violaxanthin, a compound which was not detected in WT fruit. Both phytoene and lutein were present at lower levels in the transgenic fruit than in the WT (by 0.4 and 0.8 fold, respectively), while the α-carotene content was 3.4 fold higher. The ABA content in *Lycb-1* fruit was lower than in the WT fruit at all three developmental stages sampled (Table 1). The over-expression of *Lycb-1* had no discernible effect on the carotenoid content of the leaf (Table 2). The formation of carotenoid uses sugar as early carbon source precursors in the biosynthetic pathway, because of the unexpected increase of the total carotenoid content, we analyzed the sugar contents. Results showed that the sucrose content was unaffected by the presence of the transgene at all three development stages, but the concentration of both fructose and glucose was lower in the *Lycb-1* than in the WT fruit at all stages, especially at the ripe stage (Fig. 1).

**Table 1:** Carotenoids contents in fruit of *Lycb-1* transgenic and wild-Type plants during development and ripening.

	Carotenoids Contents (µg/g DW)/ABA Contents (ng/g FW)					
	Mature green		Breaker		Ripe	
Carotenoid	Wild type	LYCB1-T_2_17	Wild type	LYCB1-T_2_17	Wild type	LYCB1-T_2_17
Phytoene	NA	NA	51.76±16.36	19.35±2.25**	666.16±38.33	493.33±38.76**
Lycopene	NA	NA	93.81±21.75	NA	913.69±100.85	809.91±75.07
α-carotene	NA	NA	NA	NA	3.68±1.00	16.09±0.54**
β-carotene	18.10±3.43	21.81±3.61	78.55±12.98	119.96±13.40*	214.01±11.75	1105.17±24.80**
Lutein	49.85±4.42	23.61±3.82**	70.07±10.72	34.99±3.04**	131.41±11.11	73.68±6.66**
Violaxanthin	NA	NA	NA	NA	NA	7.68±2.64**
ABA	164.88±9.02	129.92±6.01**	516.27±10.53	377.06±10.28**	496.02±13.89	399.48±10.90*

Mature green, 37±1 day post anthesis; Breaker, 44±1 day post anthesis; Ripe, 10 day post breaker. Data presented as the average with SD of 3 repeats, Student's t tests illustrate statistical significance (* P<0.05, ** P<0.01). DW: dry weight; FW: fresh weight; NA: not applicable.

**Table 2:** Carotenoids contents in the leaf of the *Lycb-1* transgenic and the wild-Type.

	Carotenoid Contents (µg/g FW)	
	Leaves	
Carotenoid	Wild type	LYCB1-T_2_17
α-carotene	1.32±0.17	1.27±0.05
β-carotene	169.05±42.40	162.40±8.11
Lutein	305.88±6.83	268.61±63.75
Violaxanthin	6.52±0.66	8.11±2.22

Data presented as the average with SD of 3 repeats. FW: fresh weight.

### Effect of the constitutive expression of Lycb-1 on the expression of other carotenogenic genes

**Figure 2:**
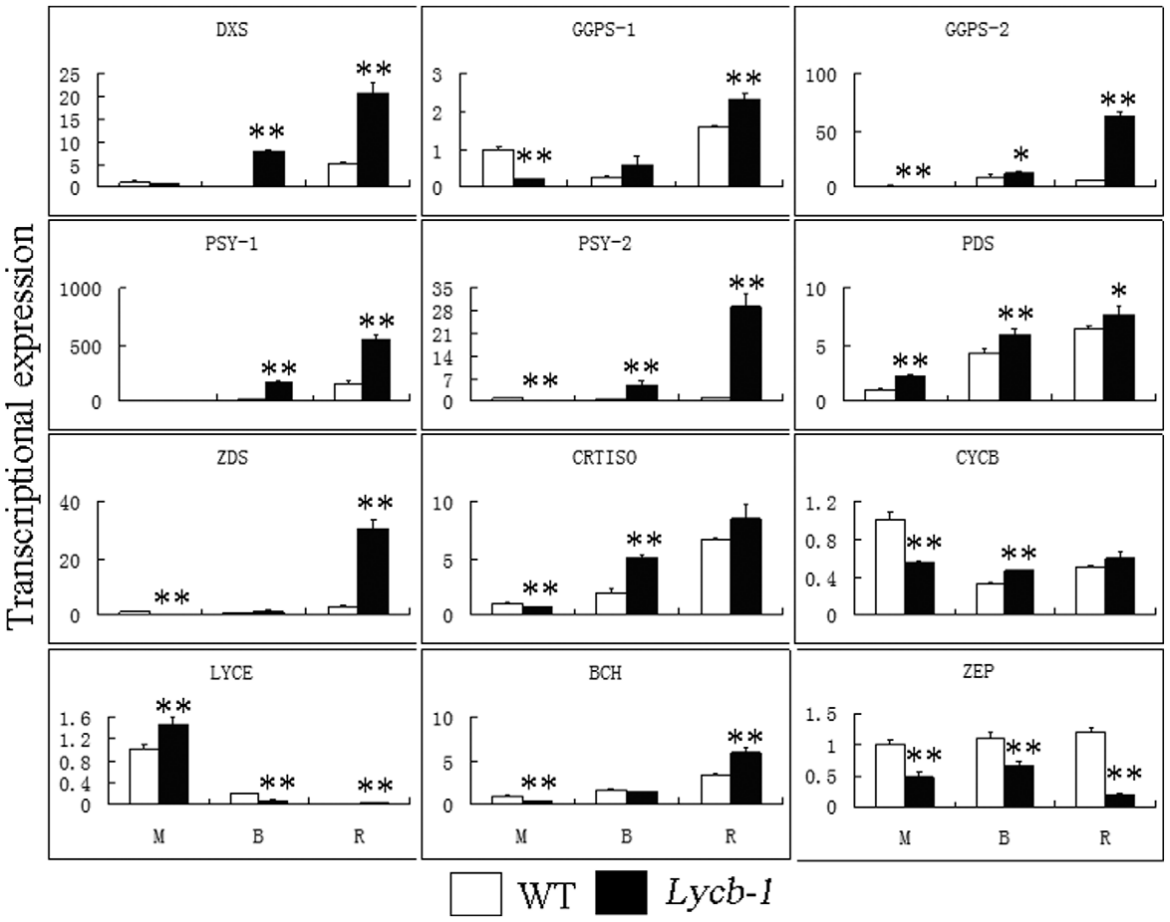
Carotenoid gene expression levels during fruit development as affected by the constitutive expression of *Lycb-1*. Columns and bars represent, respectively, the mean and SD (n = 4), Student's t tests illustrate statistical significance (* P<0.05, ** P<0.01). M: mature green, B: breaker, R: ripe.

The expression levels in both *Lycb-1* and WT of the genes *Dxs* (deoxyxylulose 5-phosphate synthase), *Ggps-1* (geranylgeranyl diphosphate synthase 1), *Ggps-2* (geranylgeranyl diphosphate synthase 2), *Psy-1* (phytoene synthase 1), *Psy-2* (phytoene synthase 2), *Pds* (phytoene desaturase), *Zds* (ζ-carotene desaturase), *Crtiso* (carotenoid isomerase), *CycB* (chromoplast specific lycopene β-cyclase), *LycE* (lycopene ε-cyclase), *Bch* (β-carotene hydroxylase) and *Zep* (zeaxanthin epoxidase) were assessed by qRT-PCR (Fig. 2). At the mature green stage, except for *Pds* and *LycE*, the transcript abundance of all of these genes was lower (between 20% and 87%) in the transgenic than in the WT fruit. At the breaker stages, all of the upstream genes (*Dxs*, *Ggps-1*, *Ggps-2*, *Psy-1*, *Psy-2*, *Pds*, *Zds*, *Crtiso*, *CycB*) were unregulated by the constitutive expression of *Lycb-1*, while transcript abundance for the three downstream genes (*LycE*, *Bch* and *Zep*) were all lower in *Lycb-1* than in WT. At the ripe stage, all but *Zep* were up-regulated in the transgenic fruit. Thus, *Zep* appeared to be down-regulated by the constitutive expression of *Lycb-1* throughout fruit development.

### Effect of the constitutive expression of Lycb-1 on the global transcriptome

**Figure 3:**
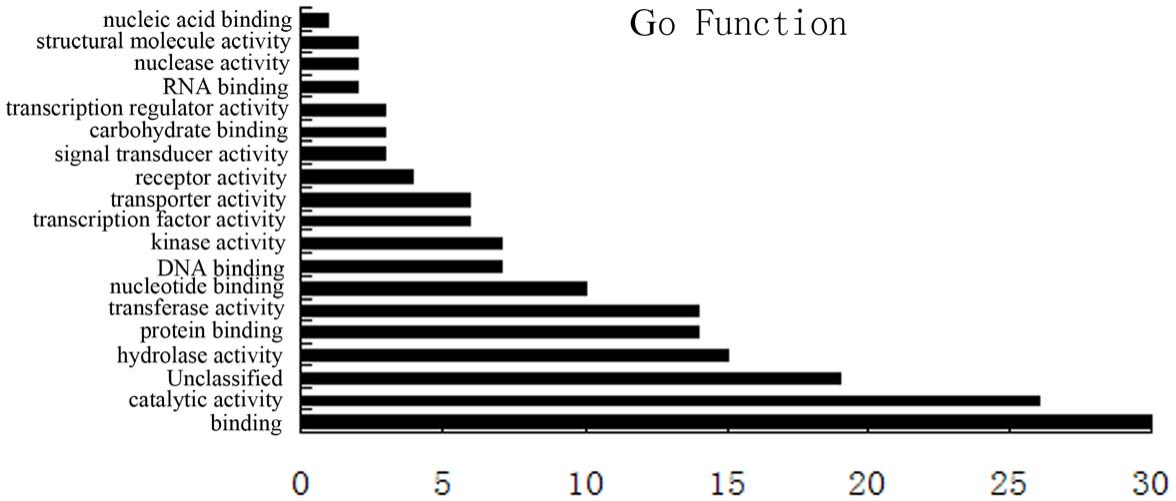
Functional categories of genes differentially expressed in the fruit of the Lycb-1 transgenic plant.

In order to investigate the impact of overexpressed *Lycb-1* gene on the global transcriptome, we compared the gene expression profile of the *Lycb-1* lines with that of the wild type using the tomato TOM2 long oligo array. Transcripts of 5729 endogenous genes showed differential expression, among which 2797 were up-regulated and 2932 were down-regulated. 93 unigenes was identified at least two fold difference in expression level in *Lycb-1* compared to WT fruit (30 up-regulated, 63 down-regulated) (Table S1) at the ripe stage. The TFGD-based assignment to GO category classified these genes into 19 categories (Fig. 3), of which the most highly represented were concerned with binding, catalytic activity, hydrolase activity, transferase activity, protein binding and nucleotide binding. A total of 17 biochemical pathways were affected by the constitutive expression of *Lycb-1*. These pathways were responsible for the synthesis or degradation of secondary metabolites, fatty acids, starch and sucrose, and for photorespiration (Table 3).

**Table 3:** Metabolic pathways identified on the basis of representation by at least three differentially expressed genes.

KEGG^a^ pathway	Genes^b^	Gene ID	Gene description	Best E-value
Fatty acid biosynthesis	3	SGN-U213509, SGN-U215683, SGN-U217263	Putative short-chain type alcohol dehydrogenase, Tropinone reductase homolog, Tropinone reductase	1.00E-31
Flavone and flavonol biosynthesis	3	SGN-U212684, SGN-U215220, SGN-U229259	CYP81B2v2, CYP92B2v1, Tetrahydroxychalcone 2′-glucosyltransferase	1.00E-51
Flavonoid biosynthesis	3	SGN-U212684, SGN-U215220, SGN-U219631	CYP81B2v2, CYP92B2v1, Gibberellin 20-oxidase-3	8.00E-55
Limonene and pinene degradation	6	SGN-U212684, SGN-U213878, SGN-U215025, SGN-U215220, SGN-U217123, SGN-U224084	CYP81B2v2, CYP72A58, Cytochrome P450-dependent fatty acid hydroxylase, CYP92B2v1, Cytochrome P450, CYP71AU1	2.00E-82
Phenylpropanoid biosynthesis	8	SGN-U212684, SGN-U213352, SGN-U213878, SGN-U215025, SGN-U215220, SGN-U215393, SGN-U217123, SGN-U224084	CYP81B2v2, Peroxidase 4, CYP72A58, Cytochrome P450-dependent fatty acid hydroxylase, CYP92B2v1, Suberization-associated anionic peroxidase 2, Cytochrome P450, CYP71AU1	4.00E-96
Photosynthesis - antenna proteins	6	M14444, SGN-U218911, SGN-U218912, SGN-U218921, SGN-U232086, SGN-U234086	Chlorophyll a/b-binding protein, Chlorophyll a/b-binding protein 3B precursor, Chlorophyll a/b-binding protein 3C, Chlorophyll a/b-binding protein 3C, like, Chlorophyll a/b-binding protein CP24 10A, Chlorophyll a/b-binding protein 2	1.00E-133
Starch and sucrose metabolism	3	SGN-U212827, SGN-U213818, SGN-U215015	Glucan endo-1,3-beta-glucosidase B, Glycoside hydrolase family 28 protein, Mannan synthase	8.00E-86

a KEGG = Kyoto encyclopedia of genes and genomes.

b The level of expression in the *Lycb-1* transgenic and Wild-Type differed significantly (p<0.05).

**Figure 4:**
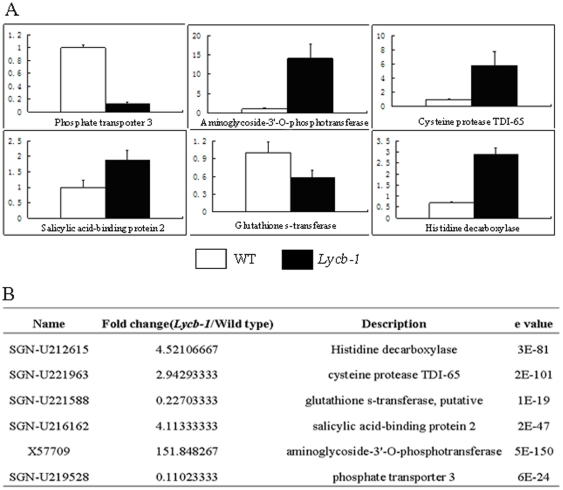
Verification of microarray results by Real-time quantitative RT-PCR. (A) Real-time quantitative RT-PCR analysis of six selected genes in Wild-Type and *Lycb-1* fruit. Columns and bars represent, respectively, the mean and SD (n = 4). (B) Expression ratios of the six selected genes derived from the microarray analysis.

RT-PCR was conducted on six of the 93 differentially expressed genes identified by the microarray analysis for validation purposes (Fig. 4). This procedure indicated a good level of consistency between the two methods of assessing gene expression.

## Discussion

We here reported the overexpression of a citrus *Lycb-1* in tomato and detected its effect on carotenoid metabolism during fruit development, as well as the effect on the transcriptome as a whole at the ripe stage.

### Flux through the α-branch of the metabolic pathway

The relative activity of *Lycb-1* and *Lyce* has been proposed as representing a possible control mechanism over the partition of flux into the β- and α-branches (end products are lutein and α-carotene respectively) of the carotenoid pathway [24], [25]. In our study, the *Lycb-1* transgenic line affects the level of LYCB present, as a result, β-branch carotenoids were accumulated, while the presence of α-branch ones declined, at least in the mature green and breaker stages of fruit development. (α-carotene accumulated at the ripe stage.) The α-carotene was repressed only when its substrate lycopene became limiting in the *Lycb-1* transgenic. During the mature green and breaker stages, little lycopene was accumulated in the fruit. In the transgenic fruit, the higher activity of LYCB presumably led to a greater channeling of lycopene through the β-branch of carotenoid synthesis, depleting the level of activity in the α-branch. By the time the ripe stage was reached, competition for lycopene had weakened, and the flux through the carotenoid pathway would have accordingly increased, thereby producing an increase in the content of α-carotene. In a related over-expression transgenic line, in which flux through the carotenoid pathway was enhanced, α-carotene were produced at a higher level than in the wild type; however, when the flux through the carotenoid pathway was not increased, the content of α-carotene decreased [26].

### Total carotenoid content increased in LYCB transformants

The constitutive expression of a gene encoding a citrus *Lycb-1* produced a notable increase in fruit carotenoid content. The effect of heterologous expression of LYCB on total carotenoid is still controversial. In tomato, heterologous expression of LYCB from *Erwinia herbicola* depressed carotenoid content, while expression of a daffodil LYCB gene strongly elevated it [20]. In our case, the increased carotenoid content may be partially explained by up-regulation of upstream flux. Phytoene synthase, encode by *Psy* gene, is the rate-limiting enzyme in the carotenoid biosynthesis pathway, many research had demonstrated that manipulation of *Psy* expression enhance carotenoid synthesis by directing metabolic flux into the carotenoid biosynthesis pathway [17], [27], [28]. The upstream pathway enzyme DXS has also been shown to increase carotenoid biosynthesis [29]. Studies based on transgenic potato tubers have demonstrated that altering the expression of certain carotenoid genes has a pronounced effect on carotenoid synthesis [28], [30]. Thus, an increase in either *Psy* and/or *Dxs* expression may underlie, at least in part, the observed rise in the carotenoid content of the fruit of the *Lycb-1* transgenic.

Another possible reason for the increased total carotenoid in LYCB transformant may be the down regulation of genes involved in downstream flux. *Zep* level was consistently down-regulated by the constitutive expression of *Lycb-1*. ZEP is an important component for the conversion of carotenoids to ABA. The ABA content in the fruit of the *Lycb-1* transgenic was much lower than that in the WT fruit across all three development stages assessed (Table 1), a behavior which is consistent with the expression profile of *Zep*. The suggestion is therefore that one effect of *Lycb-1* over-expression is that the rate of carotenoid degradation was reduced. A reduction in *Zep* expression has also been reported to produce a major increase in the carotenoid content of the potato tuber [31], [32]. The synthesis of carotenoids requires sucrose, glucose and fructose as early precursors, so starch and sucrose metabolism may play a part in increasing total carotenoid content. Note that sucrose content was unaffected by the presence of the transgene, although that of both glucose and fructose was decreased, indicating a higher rate of consumption of the two monosaccharides and an increased flux into the carotenoid synthesis pathway. A reasonable working hypothesis is that the enhanced carotenoid accumulation taking place in *Lycb-1* fruits is due to a combination of increased metabolic flux into the carotenoid synthetic pathway and the transcriptional control of a number of carotenoid metabolism genes.

### Other pathways affected by the constitutive expression of Lycb-1

The microarray data showed that some biochemical pathways genes were affected by the constitutive expression of *Lycb-1*, similarly 45% metabolites were also effected in *Psy-1* transgenic tomato fruits [33], while little effects had been observed in the *crtB* transgenic potato tubers [34]. Compare these studies with our study may indicated that the transgenic effects of carotenoid genes are different among species. The tomato fruits are rich in carotenoids while the potato tubers have little, we hypothesized that the effect of expression a carotenoid biosynthetic transgene on the endogenous pathway genes are larger in tomato fruits than that in potato tubers. The expression level of eight genes involved in phenylpropanoid synthesis was significantly changed by *Lycb-1* over-expression. Two of these genes encoding the peroxidase were significantly down-regulated, which mediated the conjugation of glutathione to unsaturated phenylpropanoids, other genes are all belong to cytochrome P450 family, which play an important role in catalyzing the phenylpropanoid synthesis pathway. Three genes involved in flavone and flavonol synthesis were down-regulated by at least two fold. One of these encodes tetrahydroxychalcone (*THC*) 2′-glucosyltransferase which catalyzes the step of the flavones branch of THC 2′-glucoside biosynthesis. The expression of three genes associated with fatty acid synthesis was also significantly changed. These genes encode the enzyme short-chain type alcohol dehydrogenase and tropinone reductase are all belong to the short-chain dehydrogenase/reductase family [35], which had been proved involved in the abscisic acid biosynthesis [36]. A total of six genes encoding photosynthesis-antenna proteins were transcriptionally at least twofold lower in the transgenic lines than in the WT lines. These genes are all belong to the chlorophyll a/b-binding protein family. The expression of three genes involved in starch and sucrose metabolism was down-regulated in *Lycb-1*; one of these encodes the enzyme glucan endo-1,3-β-glucosidase which play an important role in catalyzing 1,3-β-glucan into D-glucose, one a member of the glycoside hydrolase family involves into the interconversion of pentose and glucuronate, and the third a mannan synthase involves into the cellulose synthesis. A pyruvate kinase isozyme A (which catalyzes pyruvate metabolism) was also down-regulated by more than two fold in the *Lycb-1* transgenic fruit. One of gene encoding the enzyme 3-hydroxyacyl-CoA dehyrogenase (a key component of fatty acid elongation) was significantly down-regulated, so fatty acid synthesis may be compromised in the transgenic line.

## Materials and Methods

### Generation of tomato material

The tomato cultivar Zhongshu No. 5 was transformed via agroinfection, using the methods described as [37]. The Agrobacterium tumefaciens strain used was EHA 105, kanamycin resistance was employed as a selection marker, and the CaMV 35 S promoter provided the means to induce constitutive expression of the entire 1.2 kb *Lycb-1* open reading frame. The wild type cultivar and its derived *Lycb-1* over-expressor have been designated WT and *Lycb-1*, respectively. Plants were glasshouse-grown under supplementary lighting, with full watering and fertilization given as required. PCR validation of transformation events were based on DNA extracted from young leaf material, as described in [38]. In brief, young leaves were full ground in a mortar and pestle under 200 µl lysis buffer, after that 550 µl lysis buffer was added and vortexes for 1 min. Then, Incubate in 65°C waterbath for 60 min. Fill the tube with chloroform/isoamyl alcohol (600 mL; 24∶1 v/v) and vortexed for 3 min. Samples were centrifuged at room temperature for 5 min at 10,000 rpm. Pipet off aqueous phase (usually approximately 0.5 mL) was transferred into new microfuge tubes. Add 2/3 to 1 times the volume of cold isopropanol to the tube. Immediately spin at 10,000 rpm for 5 min. The nucleic acid pellets was washed with 75% ethanol by volume, air dried, resuspended in 50 µl of TE. PCR was performed using the following program: denaturation, 94°C for 1 min; annealing, 57°C for 1 min; extension, 72°C for 1.5 min; number of cycles, 30; final extension, 72°C for 10 min.Southern blotting experiments were based on standard procedures. Genomic DNA (20 to 30 mg) was digested with *Eco*RV. Digested, genomic DNA fragments were separated on 0.8% agarose gels and then blotted onto membrane and hybridized at 65°C overnight. Blots were probed with a PCR-derived *npt*II fragment. Membranes were washed twice with 2×SSC (0.15 M NaCl, 15 mM citric acid, and 0.2% SDS) at room temperature and then once with 2×SSC, 0.1% SDS at 65°C before film exposure.

### Experimental design

**Figure 5:**
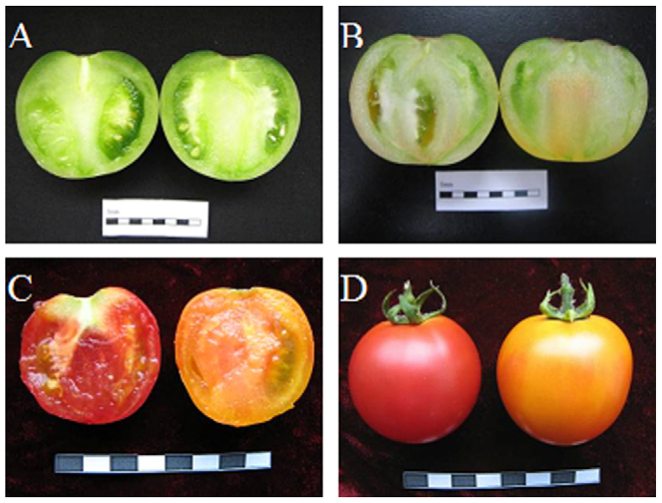
Development stages sampled in the fruits of Wild-Type (left) and *Lycb-1* transgenic (right) plants. (A) mature green, (B) breaker, (C, D) ripe.

The transgenic and its wild type plants (each fifteen) were glasshouse grown under supplementary lighting. Plants were watered as needed and fertilized following the manufacturer's directions. The *Lycb-1* and its wild type fruits at mature green, breaker, and ripe stages (Fig. 5) were collected. Immediately upon harvesting, fruit were halved and deseeded, immediately frozen in liquid nitrogen and stored at −80°C until analysis. Three biological pools of ripe stage fruit were used for the Tom2 microarray analysis, one biological pool was a mixture of four to five individual fruits from three separate plants. For other analysis, at least ten fruits from six plants were cut into small pieces and pooled. One aliquot was used for gene expression analysis, and four technical repeats were performed. Three aliquots of fruit samples were used for metabolism determination.

Statistical tests were performed using SAS software (SAS Institute, Inc., Cary NC, USA). Pooled samples were averaged and the dates were analyzed using the Student's t tests, which used to determine significant differences (* p<0.05, **p<0.01) between pairwise comparison of the wild type and the *Lycb-1* lines.

### RNA extraction

Total RNA was isolated using the TRIzol reagent (Invitrogen, USA) according to the manufacturer's instructions. RNA quality was monitored by gel electrophoresis and the measurement of the A260/A280 ratio.

### TOM2 microarray analysis

Transcriptomic profiles of *Lycb-1* and WT ripe fruit were obtained by using the TOM2 long oligo (http://ted.bti.cornell.edu/) microarray. The analysis included three biological replicates. Total RNA (5 µg) and T_7_-Oligo (dT)_15_ (Boya, China) was employed to reverse transcribe double-stranded cDNA, using a DNA Synthesis kit (Promega, USA), and then transcribed into cRNA using the T_7_ RiboMAX Express Large Scale RNA Production System (Promega, USA). A 2 µg aliquot of cRNA was combined with random primer 9 (New England Biolabs, USA) and 200 µ/µl M-MLV (Invitrogen, USA) to synthesize produce cDNA. Finally, a 2 µg sample of the cDNA was combined with Klenow polymerase (Takara, Japan), random primer 9, and final concentrations of 120 µM dATP, dGTP and dTTP, 60 µM dCTP and 40 µM of both Cy5- and Cy3-dCTP, to produce Cy5/Cy3-labelled cDNA. The labelled cDNA was hybridized overnight with the microarray at 42°C. Subsequently, the arrays were washed with 0.2% w/v SDS and 2×SSC at 42°C for 5 min, followed by washing with 0.2% SSC for 5 min at room temperature. A Printtip Lowess Normalization strategy was applied to normalize the ratio values for each array using the marray package in Bioconductor [39]. The arrays were scanned with a LuxScan 10KA confocal laser scanner (CapitalBio, China), and the resulting images analyzed by ImaGene image analysis software (BioDiscovery, CA, USA). Spots with mean signal intensity below that of the local background plus two standard deviations in both channels were regarded as being empty, and were not included in the statistical analysis. Sequences associated with a false discovery rate of <0.05 and a fold change >2 were considered as representing differentially expressed genes. The microarray hybridization raw data have been deposited at the GEO under accession number GSE33435; we can confirm all details are MIAME compliant. The Tomato Functional Genomics Database (TFGD) (http://ted.bti.cornell.edu/) was used to identify significantly affected biochemical pathways and highly enriched GO terms, as well as the functional classification of differentially expressed genes [40].

### Real-time quantitative RT-PCR (qRT-PCR)

Primer pairs were designed to amplify various gene fragments using Primer Express software (Applied Biosystems, Foster City, CA, USA). The tomato elongation factor 1α (EF-1α) was used as a reference sequence. Each 20 µl reaction contained the relevant primers (one pair directed at the gene target, and one at the reference sequence. Primer sequences given in Table 4) in SYBER GREEN PCR Master Mix (PE Applied Biosystems), and was run on an ABI 7500 Real Time System device (PE Applied Biosystems, Foster City, CA, USA). The reaction profile comprised an initial denaturation of 50°C/120 s and 95°C/60 s, followed by 40 cycles of 95°C/15 s, 60°C/60 s.

**Table 4:** Gene-specific primer sequences used for qRT-PCR.

Gene	Forward Primer (5′-3′)	Reverse Primer (5′-3′)	Accession number
DXS	TGCATTTCCTGGGATTTTGAA	TGAATCCATCCAGAGAACAAAGG	AF143812
GGPS-1	GACAGCATCTGAGTCCGTCA	CTTGGCCAGGACAGAGTAGC	U215952
GGPS-2	GGGATTGGAAAAGGCTAAGG	AGCAATCAATGGAGCAGCTT	SGN-U223568
PSY-1	CAAATGGGACAAGTTTCATGGA	TTCCTATGCCTCGATGAATCAA	Y00521
PSY-2	CACTAGCAAAGACATGAATGAAGTTTC	AACACATGTAGCAAAGATAGCTCATTTAC	L23424
PDS	AAGGCGCTGTCTTATCAGGAAA	TAAACTACGCTTGCTTCCGACA	X59948
ZDS	ACCGTACAACTACGCTACAATGG	CATCTGGCGTATAGAGGAGATTG	AF195507
CRTISO	CCCAGGGCTTAAGTCATCTATTC	GGTCCATAGGTACCACTATCACG	AF416727
LYCB	GGACCCCATTTGAAGTTTTC	AACCATGATGTGGGTTCAGA	X86452
CYCB	CTTTTCGGACATGGCTCAAAC	GCTAGATTGCCAATCAGTCTAACCA	AF254793
LYCE	GCAGGGATTTCTTGGTTCAAGT	TGATCAAGGCCTTTTCTCATGTCA	Y14387
BCH	GCTACATTCACTCTCTCGTTTGG	AAGGTCCTTCTCTTGGTCTATGG	Y14810
ZEP	CCTTGTAGGAGCTTGGAAAATG	CCCCAGAGTCAGTCTTCTCTGTA	Z83835
EF-1α	AGATGGTCAGACCCGTGAAC	TGGAGTACTTGGGGGTGGTA	X14449

### Extraction and quantification of carotenoid, ABA and sugar contents

Carotenoid pigments were extracted and then analyzed using RP-HPLC as described in [41]. Carotenoids were eluted with MeOH-MTBE-H_2_O [81∶15∶4 (v/v), eluent A] and MeOH-MTBE-H_2_O [10∶90∶4 (v/v), eluent B] by a C30 carotenoid column from YMC (YMC, Kyoto, Japan). The linear gradient program was performed as follows: initial condition was 100% A to 100% B in 90 min, and back to the initial condition for reequilibration. Carotenoids were identified by their characteristic absorption spectra, typical retention time, and comparison with authentic standards. The HPLC grade β-carotene and lycopene standards were obtained from Sigma (St Louis, MO, USA), while phytoene, α-carotene, lutein, and violaxanthin were obtained from CaroteNature (Lupsingen, Switzerland). ABA concentration was determined by LC-MS based on a 1 g sample of powdered fruit, as described in [42] with some modifications. Samples were extracted with 400 µl of 10% methanol containing 1% acetic acid to which internal standards had been added (1 ng of ^2^H_6_ ABA), then placed on ice shake for 30 min then centrifuged at 13,000 rpm for 15 min at 4°C. The supernatant was carefully removed and the pellet re-extracted with 400 µl of 10% methanol containing 1% acetic acid. Following further 30 min incubation on ice the extract was centrifuged and the supernatants pooled. Samples (50 µl) were then analyzed by LC-MS. Soluble sugar content was determined by gas chromatography, based on a 3 g sample of powdered fruit, using a method described in [43]. Three independent extractions were performed per sample.

## Supporting Information

Figure S1 **Southern blots analysis of some of** ***Lycb-1*** **transgenic plants (EcoRV/NPTII).** 1:LYCB1-T_0_17; 2:LYCB1-T_0_18; 3:LYCB1-T_0_20; 4:LYCB1-T_0_23; 5:Marker; 6:LYCB1-T_0_24; 7:LYCB1-T_0_26; 8:LYCB1-T_0_28; 9:LYCB1-T_0_30; 10:LYCB1-T_0_32.
(TIF)

Table S1 **List of the up- and down-regulated genes in** ***Lycb-1*** **transgenic ripe fruits.**
(XLS)
