# Supplementary figures and images for: Effect of the Citrus Lycopene β-Cyclase Transgene on Carotenoid Metabolism in Transgenic Tomato Fruits
Source: PLoS One. 2012 Feb 24;7(2):e32221. doi: 10.1371/journal.pone.0032221 (PMC3286453; doi:10.1371/journal.pone.0032221)

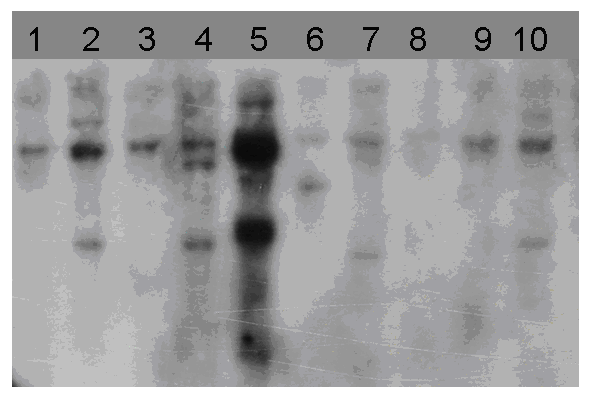

Supplement: Figure S1 — Southern blots analysis of some of Lycb-1 transgenic plants (EcoRV/NPTII). 1:LYCB1-T017; 2:LYCB1-T018; 3:LYCB1-T020; 4:LYCB1-T023; 5:Marker; 6:LYCB1-T024; 7:LYCB1-T026; 8:LYCB1-T028; 9:LYCB1-T030; 10:LYCB1-T032. (TIF) [file pone.0032221.s001.tif]
